# Amino Acid Dysregulation in the Mother–Fetus Unit: Multi-Compartment Metabolomic Signatures of Gestational Diabetes Mellitus and Fetal Macrosomia

**DOI:** 10.3390/ijms27083346

**Published:** 2026-04-08

**Authors:** Natalia A. Frankevich, Alisa O. Tokareva, Anna A. Derenko, Vitaliy V. Chagovets, Anastasia V. Novoselova, Vladimir E. Frankevich, Gennadiy T. Sukhikh

**Affiliations:** 1 National Medical Research Center for Obstetrics, Gynecology and Perinatology Named After Academician V.I. Kulakov of the Ministry of Healthcare of Russian Federation, 117997 Moscow, Russia; a_tokareva@oparina4.ru (A.O.T.); a_derenko@oparina4.ru (A.A.D.); v_chagovets@oparina4.ru (V.V.C.); a_novoselova@oparina4.ru (A.V.N.); v_frankevich@oparina4.ru (V.E.F.); g_sukhikh@oparina4.ru (G.T.S.); 2 Laboratory of Translational Medicine, Siberian State Medical University, 634050 Tomsk, Russia; 3 Department of Obstetrics, Gynecology, Perinatology and Reproductology, Institute of Professional Education, Federal State Autonomous Educational Institution of Higher Education, I.M. Sechenov First Moscow State Medical University of the Ministry of Health of the Russian Federation, 119991 Moscow, Russia

**Keywords:** gestational diabetes mellitus, fetal macrosomia, amino acid profiling, metabolomics, maternal serum, umbilical cord blood, amniotic fluid, branched-chain amino acids, metabolic pathways, biomarkers

## Abstract

The role of amino acid disturbances in the mother–fetus system remains poorly understood, despite their critical involvement in gestational diabetes mellitus (GDM), fetal macrosomia (FM) and offspring metabolic programming. This study included 62 mother–newborn dyads stratified by GDM and FM status. An analysis of the association of amino acids with clinical parameters was performed using the Spearman test. Amino acid markers of GDM were identified using the mutual information index and the Mann–Whitney test. A random forest method was used to identify amino acid markers, with the SHAP value used to estimate the contribution of each amino acid. In maternal serum, GDM was associated with significantly lower levels of glycine, 1-methylhistidine, γ-aminobutyric acid, lysine, and tryptophan. Umbilical cord serum from GDM pregnancies showed reduced concentrations of glutamine, glycine, asparagine, methionine, and proline. In amniotic fluid, GDM with FM was characterized by elevated lysine and 1-methylhistidine. Cord blood exhibited increased lysine, proline, leucine, and allo-isoleucine, while amniotic fluid showed low homocitrulline, asparagine, and lysine, together with high histidine. Fetal weight correlated directory with lysine and isoleucine and inversely with homocitrulline. Pathway analysis linked maternal serum markers to disturbances in biotin, glutamate, and carnitine metabolism, whereas cord blood markers involved broader alterations in amino acid, purine, and amino sugar metabolism. In amniotic fluid from GDM with FM, the methylhistidine pathway was specifically enriched, suggesting changes in neonatal muscle protein turnover. GDM induces distinct alterations in the amino acid profiles of all three compartments, and the combination with FM yields unique metabolic signatures. These findings identify candidate biomarkers for prediction of GDM and its complications and point to potential targets for metabolic intervention.

## 1. Introduction

Gestational diabetes mellitus (GDM) is one of the most common metabolic disorders occurring during pregnancy and is characterized by first-diagnosed hyperglycemia, the severity of which ranges from mild glucose tolerance impairment to overt diabetes requiring insulin therapy. The prevalence of this pathology is steadily increasing worldwide due to the obesity epidemic, advancing maternal age, and lifestyle changes, reaching 7% to 20% of all pregnancies across different populations [1,2,3]. Beyond its direct impact on the course of gestation, GDM is associated with a wide range of adverse maternal and perinatal outcomes, among which fetal macrosomia (FM) holds a prominent place. FM occurs in 15–45% of women with this pathology and represents a risk factor for birth trauma, operative delivery, and metabolic disorders in offspring during postnatal development [4,5,6,7].

Traditionally, research on the pathogenesis of GDM and its complications has focused primarily on carbohydrate and lipid metabolism disturbances, whereas the role of amino acids—which serve not only as building blocks for protein synthesis but also as signaling molecules modulating insulin secretion, tissue glucose sensitivity, and epigenetic processes—has been studied to a considerably lesser extent [7,8]. Amino acids play a critical role in fetal development, meeting the demand for nitrogenous compounds and participating in the formation of the nervous system, muscle tissue, and internal organs. Their transport across the placenta is mediated by specialized active transport systems, the functional activity of which may be altered under conditions of hyperglycemia and hyperinsulinemia [9,10,11]. In recent years, emerging studies have indicated associations between concentrations of specific amino acids, particularly branched-chain amino acids, and the risk of developing insulin resistance [12,13]. Comprehensive analysis of the amino acid profile in the mother–fetus system in GDM, including comparative data from maternal serum, cord blood, and amniotic fluid, has been performed in only a few studies [14,15].

Of particular interest is the investigation of amino acid status in GDM complicated by FM, since excessive amino acid supply to the fetus may represent a key factor stimulating pancreatic β-cell hyperplasia, insulin hypersecretion, and consequently, excessive adipose tissue accumulation, independent of maternal glycemia levels. Amniotic fluid, serving as an integrative compartment that reflects fetal metabolic status and excretory function, may contain unique information regarding amino acid metabolism patterns in different GDM phenotypes; however, its diagnostic potential in this context remains largely unexplored. Correlation analysis between amino acid levels in various biological compartments and clinical parameters of mothers and newborns enables not only the identification of the most informative markers but also the reconstruction of pathogenetic mechanisms underlying adverse pregnancy outcomes.

Modern multivariate statistical methods, including machine learning algorithms and SHAP value calculation, offer new opportunities for identifying metabolic signatures associated with the development of GDM and its complications, overcoming the limitations of traditional approaches based on the analysis of individual parameters. Metabolic pathway enrichment analysis using specialized databases allows us not only to detect alterations in individual amino acid concentrations but also to understand which biochemical processes are involved in disease pathogenesis—knowledge that is crucial for developing targeted therapeutic strategies.

The aim of the present study was to perform a comparative analysis of amino acid concentrations in maternal serum, cord blood, and amniotic fluid from women with physiological pregnancies and GDM, and to assess their association with the development of FM, in order to identify potential metabolic biomarkers and elucidate the pathogenetic mechanisms underlying this pathology.

## 2. Results

### 2.1. Clinical and Anamnestic Parameters

Clinical and anamnestic data were analyzed from 62 mother–newborn dyads. Comparative analysis of women with GDM (n = 37) and without GDM (n = 25) revealed a number of differences, presented in Table S1.

The median age in the GDM group was 36 years, whereas in the control group, the age was lower at 33 years. Although these differences did not reach statistical significance, they demonstrate an important clinical trend. The median age in the GDM group exceeds the 35-year threshold, which is a well-established risk factor. IVF rates were comparable between groups (8% vs. 11%, *p* = 1.00), allowing for exclusion of conception method as a confounding factor for GDM development in this cohort. High risk of preeclampsia (PE) according to first-trimester screening was detected six times more frequently (27% vs. 4% of cases) in the GDM group compared to the control group (*p* = 0.047). This finding is likely related to shared pathogenetic mechanisms between GDM and PE, including endothelial dysfunction, systemic inflammation, and insulin resistance, and supports the need for comprehensive risk assessment [16]. Pre-pregnancy BMI was significantly higher in the GDM group (24.45 kg/m^2^ vs. 22.15 kg/m^2^, *p* = 0.03). Notably, the upper quartile in the GDM group (28.72 kg/m^2^) already corresponds to overweight status. Importantly, 54% of women with GDM had normal baseline weight, indicating heterogeneity in pathogenesis and the need for universal screening. BMI at delivery was also significantly higher in the GDM group (30.09 kg/m^2^ vs. 27.89 kg/m^2^, *p* = 0.03), with the median value in the GDM group corresponding to class I obesity. Pathological weight gain was observed only in the GDM group (in 14% of women), emphasizing the importance of monitoring gestational weight gain. Family history of diabetes mellitus was identified in 43% of women with GDM (43% vs. 16%, *p* = 0.048), confirming the significant role of genetic predisposition [17]. Family history of diabetes and pre-pregnancy BMI are among the factors most strongly influencing the odds of developing GDM. The combined contribution of BMI and family history of diabetes to GDM development is presented in Figure S1.

Dietary therapy was received by 89% of women with GDM, and insulin therapy by 32%, with insulin prescribed to 19% in the second trimester and 32% in the third trimester, reflecting the natural progression of insulin resistance.

A history of macrosomia was observed only in the GDM group (16% vs. 0%, *p* = 0.09), consistent with data indicating an increased risk of recurrence. Newborn birth weight did not differ significantly between groups (3600 g vs. 3518 g, *p* = 0.37). Apgar scores at 1 and 5 min were equally high in both groups (8 and 9 points, respectively). Only newborns from the GDM group required observation and treatment in the neonatal intensive care unit (5%) and the neonatal pathology department (8%). However, these differences were not statistically significant.

Next, clinical and anamnestic data from 62 mother–newborn dyads were analyzed according to the presence of GDM and FM. The dyads were stratified into four subgroups: Subgroup 1 (GDM−, fetal normosomia, n = 18), Subgroup 2 (GDM−, FM, n = 7), Subgroup 3 (GDM+, fetal normosomia, n = 24), and Subgroup 4 (GDM+, FM, n = 13). Comparison of the four subgroups revealed a number of statistically significant intergroup differences, as well as several clinically important trends that did not reach statistical significance but warrant discussion in the context of current evidence (Table S2).

A tendency toward higher maternal age was observed in subgroups with GDM, consistent with data from the HAPO study [18], which identified age over 35 years as a risk factor for GDM and FM. IVF rates were comparable across all subgroups (*p* = 0.85), ranging from 6 to 14. Pre-pregnancy BMI differed significantly between subgroups (*p* = 0.02), reaching minimum values in Subgroup 1 (21.8 kg/m^2^) and maximum values in Subgroup 4 (26.5 kg/m^2^, *p* = 0.007). In Subgroup 4, 38% of women were overweight and 23% had class I obesity. These findings align with data published in *Lancet Diabetes & Endocrinology* regarding the association between elevated BMI and risk of GDM and FM [19], and further support the concept of GDM heterogeneity with distinct insulin-resistant and secretory subtypes. Median weight gain was 12 kg in Subgroups 1, 2, and 4, whereas Subgroup 3 had the lowest median gain at 9.5 kg (*p* = 0.16). The frequency of pathological weight gain (*p* = 0.008) was absent in Subgroups 1 and 2, was 4% in Subgroup 3, and reached 31% in Subgroup 4. This is consistent with findings from the LIFE-Moms Prospective Meta-Analysis [20], demonstrating that excessive gestational weight gain in GDM increases the risk of FM by 2.5–3-fold. The highest BMI at delivery was observed in Subgroups 2 and 4 (30.8 and 30.9 kg/m^2^, *p* < 0.001), further confirming data linking BMI > 30 kg/m^2^ to a twofold increased risk of FM. Family history of diabetes (*p* = 0.07) was observed across all subgroups, reaching 50% and 31% in Subgroups 3 and 4, respectively. Dietary therapy was prescribed to 96% of women in Subgroup 3 and 77% in Subgroup 4 (*p* = 0.03), while insulin therapy was administered to 33% and 31%, respectively (*p* = 0.02), underscoring the importance of timely treatment initiation. History of macrosomia (*p* = 0.03) was present only in GDM subgroups: in 8% of Subgroup 3 and 31% of Subgroup 4, consistent with evidence of high recurrence risk for FM [21]. Male fetal sex was significantly more prevalent in subgroups with FM, reaching 86% and 77% in Subgroups 2 and 4 (compared to 39–38% in subgroups without FM, *p* = 0.02), aligning with published data indicating an increased risk of FM in male infants [22].

Fetal weight was most strongly influenced by maternal pre-pregnancy BMI and infant sex (Figure S2), with male infants having higher birth weights than female infants born to women with equivalent BMI.

### 2.2. Amino Acid Profiling

Distribution of samples in principal component space in all fluids are associated with GDM development (Figure S3a,c,e) along the two first principal components in the case of the maternal venous serum (Figure S3a), along the first principal component in the case of the maternal umbilical serum (Figure S3c) and along the two first principal components in the case of amniotic fluid (Figure S3e).

In the case of the maternal venous serum amino acids, the most valuable amino acids for the first component (part of descripted variation 49%) were asparagine, glutamic acid, tryptophane, aspartic acid, glycine, glutamine, 1-methylhistidine, histidine, phenylalanine, and γ-aminobutyric acid, and the most valuable amino acids for the second principal component (part of descripted variation 7%) were 3-aminoisobutyric acid, α-aminoisobutyric acid, arginine and allo-isoleucine (Figure S3b).

In the case of the maternal cord serum, the most valuable amino acids for the first principal component (part of descripted variation 43%) are methionine, aspartic acid, asparagine, tyrosine, phenylalanine, glutamic acid, proline, glycine, alanine, glutamine, and γ-aminobutiric acid, and the most valuable for the second principal component (part of descripted variation 12%) are homocitrulline, β-alanine, threonine, α-aminobutyric acid, isoleucine, leucine, and valine (Figure S3d).

In the case of amniotic fluid, the most valuable amino acids for the first principal component (part of descripted variation 64%) are isoleucine, aspartic acid, asparagine, glutamic acid, serine, methionine, tyrosine, phenylalanine, glutamine, leucine, citrulline, proline, 2-aminoadipic acid, glycine, alanine, tryptophan, and threonine, and the most valuable for the second principal component space are 3-aminoisobutyric acid, homocitrulline, histidine, 1-methylhistidine and α-aminobutyric acid (Figure S3f).

Total protein and fibrinogen concentrations in maternal blood prior to delivery exhibited strong inverse correlations with amino acid levels in maternal venous serum. The strongest associations were observed between total protein and proline (R = −0.56, *p* < 0.001), tryptophan (R = −0.52, *p* < 0.001), methionine (R = −0.49, *p* < 0.001), aspartic acid (R = −0.48, *p* < 0.001), alanine (R = −0.47, *p* < 0.001), asparagine (R = −0.46, *p* < 0.001), glutamine (R = −0.45, *p* < 0.001), tyrosine (R = −0.45, *p* < 0.001), phenylalanine (R = −0.43, *p* < 0.001), isoleucine (R = −0.43, *p* < 0.001), and citrulline (R = −0.41, *p* < 0.001). Fibrinogen showed strong inverse correlations with 1-methylhistidine (R = −0.44, *p* < 0.001), histidine (R = −0.42, *p* < 0.001), and γ-aminobutyric acid (R = −0.42, *p* < 0.001) (Figure S4a). Earlier, asparagine, aspartic acid, glutamine, phenylalanine, and γ-aminobutyric acid were identified as amino acids with a large influence on the first principal component (projection on axis > 0.2) (Figure S3b).

Pre-pregnancy BMI demonstrated weak positive correlations with 3-aminoisobutyric acid (R = 0.29, *p* = 0.02). Maternal BMI at delivery showed weak positive correlations with 3-aminoisobutyric acid (R = 0.28, *p* = 0.02) and 2-aminoadipic acid (R = 0.28, *p* = 0.02). Gestational weight gain exhibited weak inverse correlations with 3-aminoisobutyric acid (R = −0.26, *p* = 0.047) and homocitrulline (R = −0.29, *p* = 0.02), and weak positive correlations with proline (R = 0.26, *p* = 0.046), tyrosine (R = 0.27, *p* = 0.03), phenylalanine (R = 0.26, *p* = 0.045), and methionine (R = 0.33, *p* = 0.008) (Figure S4a). Earlier, 3-aminoisobutyric acid was identified as an amino acid with a large influence on the second principal component and phenylalanine was identified as an amino acid with a large influence on the second principal component (projection on axis > 0.2) (Figure S3b).

Total protein and fibrinogen concentrations in maternal blood prior to delivery exhibited strong inverse correlations with amino acid levels in maternal cord serum. The strongest associations were observed between total protein and proline (R = −0.61, *p* < 0.001), alanine (R = −0.54, *p* < 0.001), glutamine (R = −0.54, *p* < 0.001), asparagine (R = −0.49, *p* < 0.001), methionine (R = −0.46, *p* < 0.001), γ-aminobutyric acid (R = −0.46, *p* < 0.001), glutamic acid (R = −0.46, *p* < 0.001), citrulline (R = −0.44, *p* = 0.002) and 1-methylhistidine (R = −0.42, *p* = 0.003). Fibrinogen showed a medium inverse correlation with serine (R = −0.46, *p* < 0.001). ALT concentration has medium direct association with concentrations of γ-aminobutyric acid (R = 0.56, *p* < 0.001), aspartic acid (R = 0.50, *p* < 0.001), β-alanine (R = 0.48, *p* < 0.001), asparagine (R = 0.47, *p* < 0.001) glutamic acid (R = 0.46, *p* < 0.001), phenylalanine (R = 0.44, *p* = 0.002), glycine (R = 0.44, *p* = 0.002), glutamine (R = 0.43, *p* = 0.003), methionine (R = 0.43, *p* = 0.003) and 1-methylhistidine (R = 0.42, *p* = 0.003) and medium inverse association with arginine concentration (R = −0.45, *p* < 0.001) (Figure S4b). Earlier, methionine, aspartic acid, asparagine, phenylalanine, glutamic acid, glycine, alanine, glutamine and γ-aminobutyric acid were identified as amino acids with a large influence on the first principal component (projection on axis > 0.2), and β-alanine was identified as an amino acid with a large influence on the second principal component (projection on axis > 0.2) (Figure S3d).

Newborn weight was weakly directly associated with concentration of allo-isoleucine (R = 0.33, *p* = 0.02), leucine (R = 0.34, *p* = 0.02), medium directly associated with concentrations of lysine (R = 0.45, *p* = 0.001) and isoleucine (R = 0.46, *p* = 0.001) and medium inverse-associated with homocitrulline concentration (R = −0.41, *p* = 0.004). Maternal BMI at delivery also was weakly inverse-associated with homocitrulline concentration (R = −0.33, *p* = 0.02). Pregnancy weight gain had an inverse association with the concentration of α-aminobutyric acid (R = −0.3, *p* = 0.04) (Figure S4b). Earlier, homocitrulline and β-alanine were identified as amino acids with a large influence on the second principal component along the axis (projection on axis > 0.2) and α-aminobutyric acid, leucine and isoleucine were identified as amino acids with a large influence on the second principal component against the axis (projection on axis < −0.2) (Figure S3d).

Gestational age at delivery and blood concentration of glucose had direct associations with the concentration of amino acids in the amniotic fluid. The strongest associations were between glucose concentration and 2-aminoadipic acid (R = 0.40, *p* = 0.005) and between gestational age of the delivery and isoleucine concentration (R = 0.41, *p* = 0.004), aspartic acid (R = 0.41, *p* = 0.004) and valine (R = 0.40, *p* = 0.005) (Figure S4c). Earlier, isoleucine, aspartic acid and 2-aminoadipic acid were identified as amniotic fluid amino acids with a large influence on the first principal component (Figure S3f)

Newborn weight had a weak direct association with amniotic fluid histidine concentration (R = 0.29, *p* = 0.046) and weak inverse association with homocitrulline concentration (R = −0.35, *p* = 0.01). Pre-pregnancy maternal BMI had a weak inverse association with homocitrulline concentration (R = −0.31, *p* = 0.03) and threonine (R = −0.29, *p* = 0.048). Pre-delivery maternal BMI also had a weak inverse association with homocitrulline concentration (R = −0.40, *p* = 0.005) (Figure S4c). Earlier, threonine was identified as an amino acid with a large influence on the first principal component (projection on axis > 0.2), and homocitrulline and histidine were identified as amino acids with a large influence on the second principal component (projection on axis > 0.2) (Figure S3f).

There is no statistically significant variation in the concentration of any amino acid in any body fluid in the case of insulin therapy compared with only diet therapy for GDM-treatment (Table S3).

### 2.3. Amino Acid Markers Search

Next, we analyzed alterations in the quantitative amino acid (AA) composition within the mother–fetus system in relation to GDM and newborn birth weight, aiming to identify potential markers of these conditions.

**Figure 1:**
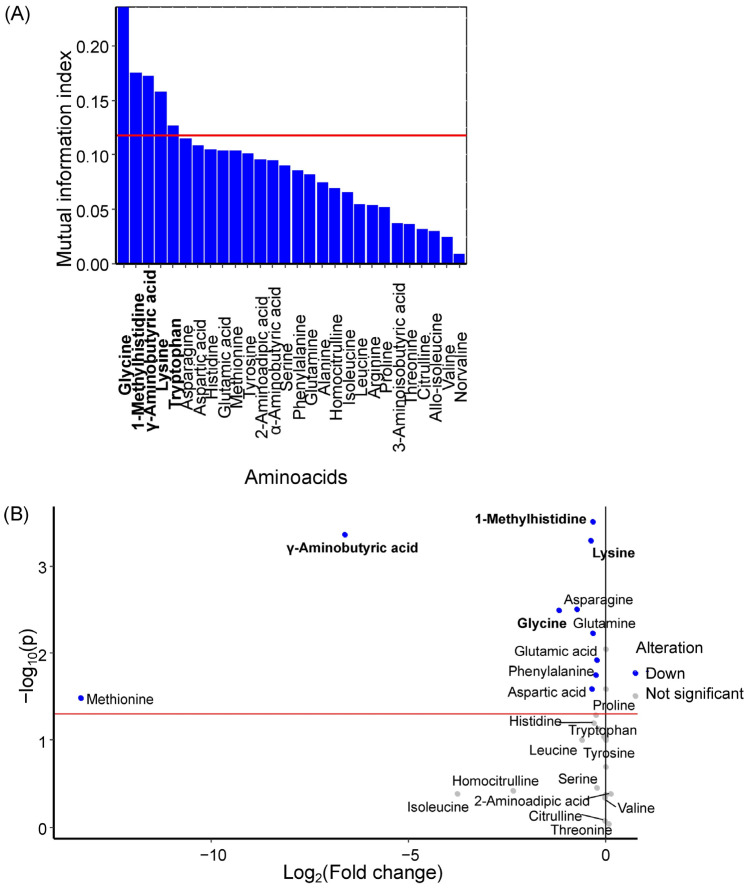
(**A**) Mutual information index between amino acid concentrations in maternal serum and the development of GDM in women. Potential marker amino acids are highlighted in bold, red line is a threshold of mutual information index for potential markers. (**B**) Volcano plot showing changes in median amino acid concentrations in maternal serum during GDM development. Marker amino acids are highlighted in bold, red line is a level of statistical significance threshold (*p* < 0.05).

Glycine, 1-methylhistidine, γ-aminobutyric acid, lysine, and tryptophan were identified as potential GDM markers in maternal venous serum (Figure 1A), with all amino acids except tryptophan showing statistically significant decreases in GDM (Figure 1B, Table 1). Earlier, tryptophan, glycine, 1-methylhistidine and γ-aminobutyric acid were identified as the some of the most important amino acids for the first principal component (Figure S3b).

**Figure 2:**
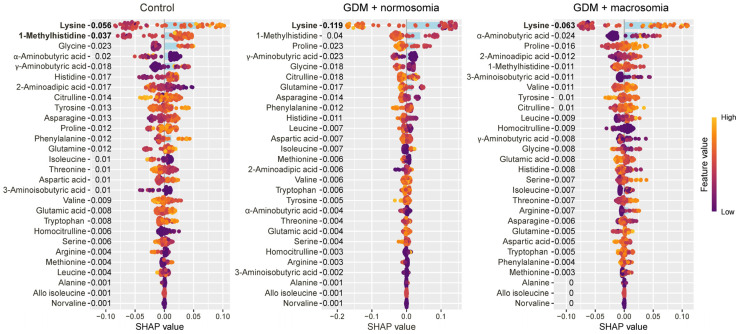
SHAP values of maternal serum amino acids for discrimination between the control group, the GDM with macrosomia subgroup, and the GDM with normosomia subgroup. Amino acids that are potential markers for each group are highlighted in bold. Numbers next to amino acids and blue bar indicate the mean absolute SHAP value, points and values on x-axis indicate SHAP value in case of each individual. Yellow represents high concentration values; purple represents low concentration values.

Lysine and 1-methylhistidine were characteristic of the control group, with lysine showing a specific decrease in GDM pregnancies resulting in normal-weight newborns (Figure 2).

**Figure 3:**
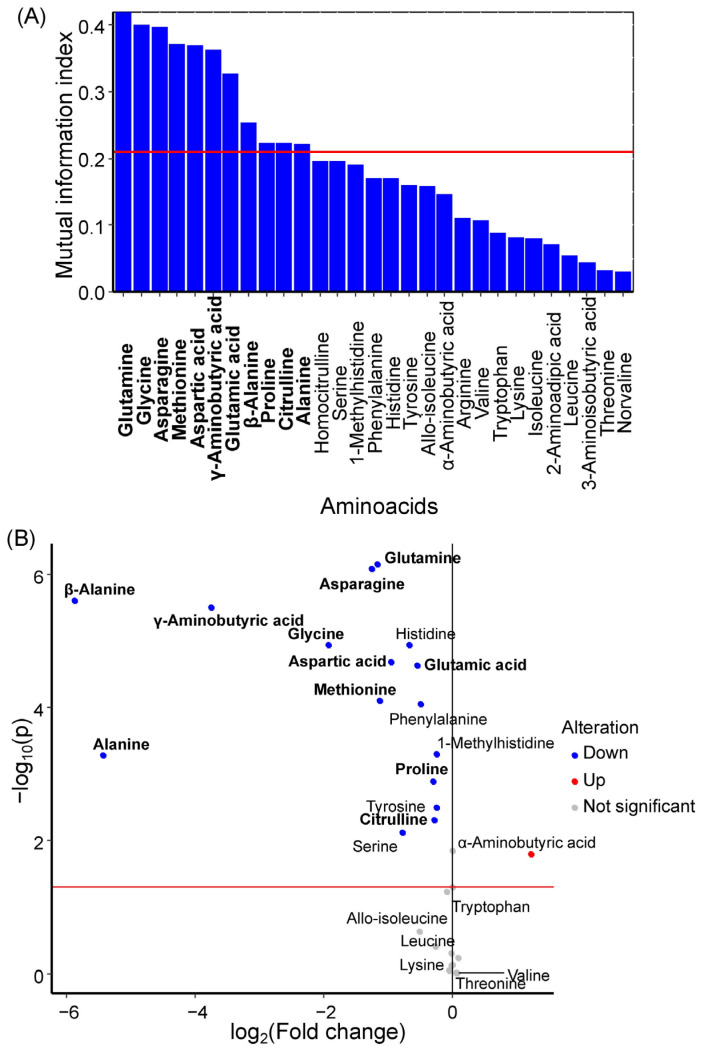
(**A**) Mutual information index between amino acid concentrations in umbilical cord serum and the development of GDM in women. Potential marker amino acids are highlighted in bold, red line is a threshold of mutual information index for potential markers. (**B**) Volcano plot showing changes in median amino acid concentrations in umbilical cord serum during GDM development. Marker amino acids are highlighted in bold, red line is a level of statistical significance threshold (*p* < 0.05).

Glutamine, glycine, asparagine, methionine, aspartic acid, γ-aminobutyric acid, glutamic acid, β-alanine, proline, citrulline, and alanine were identified as potential GDM markers in umbilical cord serum (Figure 3A). All of these amino acids exhibited statistically significant decreases in concentration during GDM development (Figure 3B, Table 2). Earlier, methionine, aspartic acid, asparagine, glutamic acid, proline, glycine, alanine, glutamine and γ-aminobutiric acid were selected as umbilical serum amino acids with the large influence on the first principal component (Figure S3d).

**Figure 4:**
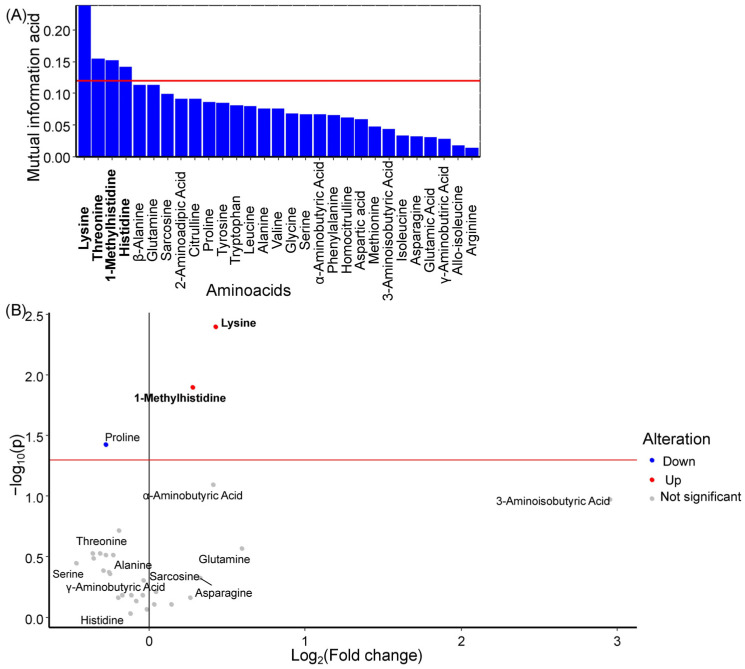
(**A**) Mutual information index between amino acid concentrations in amniotic fluid and the development of GDM in women. Potential marker amino acids are highlighted in bold, red line is a threshold of mutual information index for potential markers. (**B**) Volcano plot showing changes in median amino acid concentrations in amniotic fluid during GDM development. Marker amino acids are highlighted in bold, red line is a level of statistical significance threshold (*p* < 0.05).

Lysine, 1-methylhistidine, and histidine were identified as potential GDM markers in amniotic fluid (Figure 4A). Among these, lysine and 1-methylhistidine levels showed statistically significant increases during GDM development (Figure 4B, Table 3). Earlier, threonine was selected as an amniotic fluid amino acid with a large influence on the first principal component and lysine, histidine, and 1-methylhistidine were selected as amniotic fluid amino acids with a large influence on the second principal component (Figure S3f).

**Figure 5:**
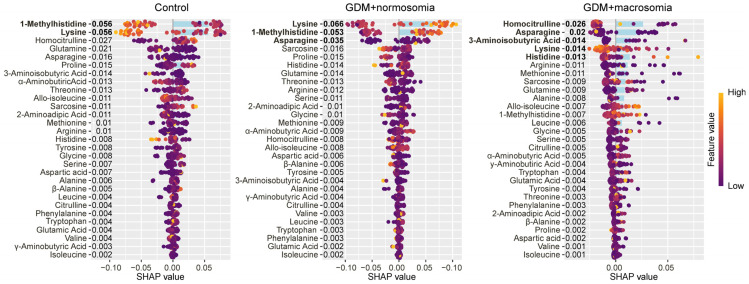
SHAP values of amniotic fluid amino acids for discrimination between the control group, the GDM with macrosomia subgroup, and the GDM with normosomia subgroup. Amino acids that are potential markers for each group are highlighted in bold. Numbers next to amino acids and blue bar indicate the mean absolute SHAP value, points and values on x-axis indicate SHAP value in case of each individual. Yellow represents high concentration values; purple represents low concentration values.

Decreased levels of lysine and 1-methylhistidine characterized the control group, whereas increased levels of these amino acids characterized the GDM group with normal-weight newborns. For GDM with normal-weight newborns, low levels of homocitrulline, asparagine, aminobutyric acid, and lysine, along with high levels of histidine, were characteristic (Figure 5).

**Figure 6:**
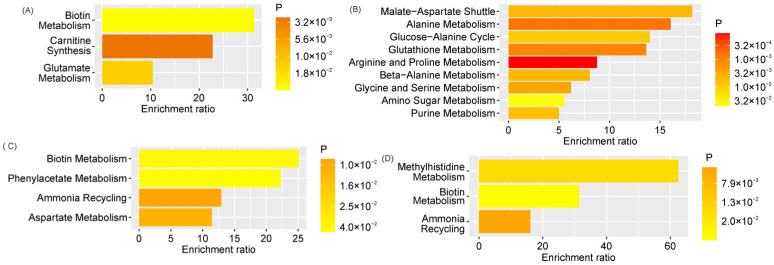
Statistically significantly enriched pathways according to the SMPDB for markers of: (**A**) GDM in maternal venous serum, (**B**) GDM in umbilical cord serum, (**C**) GDM in amniotic fluid, and (**D**) GDM with macrosomia in amniotic fluid.

GDM markers in maternal venous serum significantly enriched pathways related to biotin metabolism, glutamate metabolism, and carnitine synthesis (Figure 6A), whereas GDM markers in cord blood predominantly enriched pathways associated with amino acid metabolism, purine metabolism, and amino sugar metabolism (Figure 6B). GDM markers in amniotic fluid significantly enriched pathways related to biotin metabolism, phenylacetate metabolism, aspartate metabolism, and the ammonia cycle (Figure 6C). Similarly, markers of GDM with macrosomia significantly enriched ammonia cycle and biotin metabolism pathways. Additionally, the methylhistidine metabolism pathway was significantly enriched (Figure 6D).

## 3. Discussion

The main risk factors for GDM in the studied cohort were high risk of PE (OR ≈ 6.75), family history of diabetes (OR ≈ 3.9), and elevated pre-pregnancy and delivery BMI. The heterogeneity of GDM is supported by the substantial proportion of women with normal weight (54%). Shared pathogenetic mechanisms with PE, evidence of systemic inflammation, and residual metabolic imbalance despite treatment underscore the need for improved therapeutic strategies. The absence of differences in most obstetric outcomes reflects the effectiveness of timely diagnosis and therapy.

These findings are fully consistent with the contemporary concept of GDM as a heterogeneous condition requiring a personalized approach. The Hyperglycemia and Adverse Pregnancy Outcome (HAPO) study [23] demonstrated that the long-term consequences of GDM for mothers (risk of type 2 diabetes mellitus, cardiovascular disease) and offspring (obesity, impaired glucose tolerance) depend not only on the degree of hyperglycemia but also on baseline metabolic status, gestational weight gain, and genetic factors. The differences observed between subgroups 3 and 4 in the present study confirm the existence of distinct GDM phenotypes: “compensated” (with weight control and dietary therapy) and “decompensated” (with pathological weight gain, elevated glucose levels, and FM). This aligns with recent work in this area [24,25].

Current international guidelines (NICE, ACOG, FIGO) emphasize the need for a comprehensive approach to managing pregnant women with GDM, including not only glycemic control but also monitoring of gestational weight gain, screening for urinary tract infections, assessment of coagulation status, and consideration of risk factors such as history of macrosomia and family history of diabetes. The data obtained in this study confirm the importance of each of these components and justify the need for further prospective studies with larger sample sizes to develop more accurate prognostic models and therapeutic strategies.

This study revealed significant differences in the amino acid profiles of maternal serum, umbilical cord blood, and amniotic fluid between GDM and physiological pregnancies, and established an association between these alterations and the development of FM, opening new perspectives for understanding the pathogenesis of metabolic disturbances and their prediction. The findings demonstrate that GDM is accompanied by profound remodeling of amino acid metabolism, affecting not only the maternal organism but also the fetoplacental unit, with the pattern of these changes differing according to the presence or absence of FM. This allows for consideration of specific amino acids as potential biomarkers for different clinical outcomes. Analysis of maternal serum identified glycine, 1-methylhistidine, γ-aminobutyric acid, lysine, and tryptophan as the most informative GDM markers, with all except tryptophan showing statistically significant concentration decreases during GDM development. This finding aligns with the current understanding of enhanced amino acid consumption by the fetoplacental complex and impaired amino acid synthesis under conditions of insulin resistance [26,27].

Of particular note is the observed differential dynamics of lysine and 1-methylhistidine, which not only decreased in GDM overall but also exhibited specific alterations in the subgroup of women delivering normal-weight newborns, indicating the existence of distinct metabolic phenotypes within the GDM group and underscoring the heterogeneity of this condition. Analysis of the amino acid profile in umbilical cord blood revealed a substantially broader spectrum of alterations, including glutamine, glycine, asparagine, methionine, aspartic acid, γ-aminobutyric acid, glutamic acid, β-alanine, proline, citrulline, and alanine, all of which also showed decreased concentrations in GDM. These processes reflect impaired placental transport and altered metabolism in fetal tissues under conditions of hyperglycemia and hyperinsulinemia [28]. Of particular interest is the identified association between levels of branched-chain amino acids, specifically leucine and allo-isoleucine, in cord blood and the development of FM, confirming the key role of these compounds in stimulating insulin secretion and activating anabolic processes in the fetal organism, leading to excessive adipose tissue accumulation and increased birth weight [14].

Analysis of amniotic fluid identified lysine, threonine, 1-methylhistidine, and histidine as GDM markers; however, a different dynamic was observed here: lysine and 1-methylhistidine levels were significantly increased in GDM, which may be related to enhanced excretion of these amino acids by the fetal kidneys or to alterations in amniotic fluid composition resulting from polyuria characteristic of fetal hyperglycemia [29,30]. Interestingly, the GDM group subsequently delivering macrosomic infants was characterized by low levels of homocitrulline, asparagine, aminobutyric acid, and lysine, alongside high histidine concentrations, whereas the opposite pattern predominated in the GDM group without macrosomia. This suggests distinct metabolic trajectories leading to excessive fetal weight gain and opens opportunities for early risk stratification.

Correlation analysis revealed a complex network of relationships between clinical parameters and amino acid profiles, with the strongest negative correlations observed between total protein and fibrinogen concentrations in maternal blood and the levels of numerous amino acids in both maternal and umbilical cord serum. This reflects the tight integration of protein and amino acid metabolism and their coordinated regulation under conditions of metabolic stress [31].

Of particular interest are the observed correlations between newborn birth weight and concentrations of specific amino acids. Interestingly, maternal BMI both before pregnancy and at delivery showed weak but statistically significant correlations with a limited spectrum of amino acids, predominantly 3-aminoisobutyric acid and 2-aminoadipic acid, whereas gestational weight gain was associated with a broader range of metabolites, including proline, tyrosine, phenylalanine, and methionine. This underscores the importance of dynamic changes in body weight, rather than baseline anthropometric measures alone, in shaping the amino acid profile [32]. Summary Table S4 presents alterations in amino acid levels across the three biological compartments (maternal serum, umbilical cord serum, amniotic fluid) in GDM, as well as features characteristic of GDM cases complicated by FM.

The metabolic pathway enrichment analysis using the SMPDB identified key biochemical processes disrupted in GDM and in GDM with FM. Markers of GDM in maternal serum were primarily associated with biotin metabolism, glutamate metabolism, and carnitine synthesis, whereas markers in cord blood enriched a broader spectrum of pathways, including amino acid metabolism, purine metabolism, and amino sugar metabolism, reflecting the systemic nature of metabolic disturbances in the fetus. In amniotic fluid, GDM markers were associated with biotin metabolism, phenylacetate metabolism, aspartate metabolism, and the ammonia cycle, whereas in GDM with FM, the methylhistidine metabolism pathway was additionally enriched. This may be directly related to altered muscle metabolism and changes in body composition in macrosomic newborns. Summary data are presented in Table S5.

The observed changes in the amino acid profiles within the maternal–fetal dyad provide critical insights into the mechanistic links between maternal metabolic dysregulation and excessive fetal growth [33]. These findings can be interpreted within the framework of key signaling pathways that integrate nutrient sensing with cellular growth and metabolism. The mammalian target of rapamycin (mTOR) pathway serves as a central nutrient sensor, particularly sensitive to amino acid availability, and plays a critical role in placental function and fetal growth [34]. Our observation of decreased branched-chain amino acid (BCAA; leucine, isoleucine, valine) levels in umbilical cord blood in gestational diabetes mellitus (GDM), alongside elevated leucine and allo-isoleucine, particularly in cases of macrosomia, is highly relevant to mTOR signaling. Leucine is a potent activator of mTOR complex 1 (mTORC1) through its interaction with sestrin 2, which relieves inhibition of mTORC1 [35]. In the placenta, mTORC1 activity regulates system A and system L amino acid transporters, establishing a positive feedback loop in which amino acid availability modulates transporter expression, which in turn determines nutrient delivery to the fetus [36,37]. The elevated leucine levels observed in fetal macrosomia may reflect hyperactivation of placental mTORC1, driving increased amino acid transport and subsequent fetal insulin secretion, thereby promoting anabolic growth. The insulin signaling pathway intersects with amino acid metabolism at multiple levels. Maternal hyperinsulinemia in GDM directly suppresses hepatic gluconeogenesis while simultaneously stimulating protein synthesis in maternal tissues, potentially contributing to the observed reduction in maternal amino acid concentrations. Fetal hyperinsulinemia, a hallmark of fetal macrosomia, further promotes amino acid uptake and protein accretion in fetal tissues [38]. Our correlation analysis, demonstrating positive associations between fetal weight and lysine (R = 0.45), isoleucine (R = 0.46), and leucine (R = 0.34), supports this mechanism. Conversely, the negative correlation with homocitrulline (R = −0.41) suggests that impaired urea cycle function may accompany excessive anabolic activity.

The amino acid profile undergoes systemic changes across compartments (mother, umbilical cord, AF); however, these changes are not mirror-like. For example, 1-methylhistidine decreases in maternal blood but increases in AF, highlighting the importance of investigating multiple compartments to understand the complete picture. Decreases in glycine and GABA indicate oxidative stress, inflammation, and impaired neuro/metabolic regulation. Reduced levels of glutamine, asparagine, and citrulline in umbilical cord blood reflect fetoplacental unit dysfunction, including impaired nutrient transport, endothelial dysfunction, and possibly intrauterine fetal metabolic stress.

Amniotic fluid serves as a unique “window” into fetal status. Alterations in AF (increased 1-methylhistidine and lysine) may reflect not so much placental transport as fetal renal function, skin and lung condition, and meconium composition [33]. This makes AF a valuable specimen for assessing the fetal response to maternal hyperglycemia. The observed changes (particularly glycine and glutamine deficiency) point to potential targets for nutritional intervention or biochemical monitoring aimed at reducing fetal risks. However, this requires further investigation.

Thus, the compartment-specific changes we observed reflect differential regulation of amino acid metabolism in maternal and fetal tissues. Decreased glycine and glutamine levels in maternal serum indicate enhanced utilization of gluconeogenic substrates and possible disruption of one-carbon metabolism, with glycine serving as a methyl donor in the methionine cycle and potentially influencing fetal epigenetic programming [39,40]. The significant enrichment of glutamate metabolism pathways among maternal markers is consistent with the role of glutamate as a key nitrogen carrier and precursor for glutathione synthesis, pointing to involvement of oxidative stress [41]. In umbilical cord blood, the widespread decrease in amino acids, including glutamine, asparagine, and methionine, suggests either impaired placental transport or enhanced fetal utilization, with methionine being critically important for methylation reactions and polyamine synthesis required for rapid fetal growth [39,42]. Enrichment of amino sugar metabolism pathways in umbilical cord blood markers may reflect alterations in glycosylation patterns or remodeling of the extracellular matrix in fetal tissues. Analysis of amniotic fluid reveals unique aspects of fetal excretory function: increased lysine and 1-methylhistidine suggests either fetal polyuria with concentration of these amino acids or altered tubular reabsorption. Histidine, which shows differential patterns in macrosomia, is a precursor of histamine and carnosine involved in antioxidant and anti-inflammatory mechanisms, and the specific enrichment of the methylhistidine metabolism pathway in GDM with macrosomia is particularly interesting, as 3-methylhistidine serves as a marker of myofibrillar protein breakdown [43], and its elevated level may reflect either increased muscle protein turnover in macrosomic fetuses or subclinical myopathy resulting from chronic hyperglycemic exposure. The distinct metabolic signatures in GDM with and without macrosomia support the concept of GDM heterogeneity: women who develop macrosomia, despite similar glycemic control, exhibit differences in amino acid metabolism that may reflect genetic variations in transporter efficiency or insulin sensitivity. Elevated branched-chain amino acid levels in umbilical cord blood in macrosomia may directly contribute to β-cell hyperplasia and hyperinsulinemia, as leucine allosterically activates glutamate dehydrogenase, potentiating glucose-stimulated insulin secretion, thereby establishing a direct link between maternal amino acid dysregulation and development of the fetal endocrine pancreas [44]. The inverse correlation of homocitrulline with fetal weight across all compartments warrants particular attention. Homocitrulline is formed by carbamylation of lysine under conditions of oxidative stress, and its low level may indicate either less oxidative protein modification or alterations in urea cycle function [45]. Amniotic fluid analysis, given its clinical accessibility, has the potential to provide prognostic information regarding the risk of excessive fetal growth. The involvement of specific metabolic pathways such as biotin metabolism, carnitine synthesis, and methylhistidine metabolism may point to potential therapeutic targets in the future, enabling a transition from mere biomarker identification to mechanistic understanding that can serve as a foundation for developing interventional strategies.

An important methodological limitation for interpretation: all conclusions drawn from this study are preliminary (hypothesis-generating) in nature due to the small sample size. They indicate interesting trends and biological mechanisms that should be confirmed in an independent, larger-scale cohort. It is important to note that in 2025, we published an article entitled “Amino Acid Profile Alterations in the Mother-Fetus System in Gestational Diabetes Mellitus and Macrosomia” [15], based on the results of a two-center study (Moscow + Tomsk) conducted from January 2024 to March 2025, which included 94 mother–fetus dyads (GDM group: 53, control group: 41; GDM + FM: 23, GDM + normosomia: 30, control: 36). Comparative analysis of data from the Tomsk patient cohort and the Moscow cohort (the present publication) revealed high consensus on key markers: glutamine, histidine, and asparagine were consistently decreased in GDM in maternal blood; isoleucine, leucine, and serine were consistently increased in FM in cord blood and AF; GABA decrease was confirmed in both studies (marker of metabolic dysregulation). Both studies confirm the involvement of amino acid transport, glutamate metabolism, and the urea cycle. Lysine and isoleucine were the most consistent positive correlates of fetal weight; homocitrulline was a consistent negative correlate of fetal weight. Both studies demonstrate high consistency of results, despite different cohorts and sample sizes. The identified markers (glutamine, histidine, asparagine in maternal blood; isoleucine, leucine, serine in cord blood and AF) can be considered valid candidates for the development of diagnostic panels. The differential dynamics of amino acids across different biological compartments underscore the need for a comprehensive approach to assessing the mother–fetus system and open prospects for early diagnosis and personalized correction of metabolic disturbances in GDM and FM.

## 4. Materials and Methods

### 4.1. Study Design

This case–control study was conducted at the Academician V.I. Kulakov National Medical Research Center for Obstetrics, Gynecology, and Perinatology, Moscow, from January to December 2025. The study included 62 mother–newborn dyads who received pregnancy care and delivered at the center. The main GDM group comprised 37 mother–newborn dyads, and the control group comprised 25 mother–newborn dyads. Inclusion criteria were: singleton pregnancy, gestational age at enrollment > 37 weeks and 1 day, absence of severe somatic pathology, and informed consent to participate in the study. Exclusion criteria included: multiple pregnancy, pregestational type 1 or type 2 diabetes mellitus, severe preeclampsia requiring preterm delivery, chronic placental insufficiency with fetal growth restriction, congenital fetal malformations, HIV infection, viral hepatitis, and refusal to participate in the study.

The diagnosis of GDM was established according to the criteria of the All-Russian Clinical Protocol “Gestational Diabetes Mellitus” (2020), based on the results of a 75 g oral glucose tolerance test performed at 24–28 weeks of gestation, or on fasting venous plasma glucose levels at the time of registration. The diagnosis was confirmed if at least one of the following threshold values was exceeded: fasting venous plasma glucose ≥ 5.1 mmol/L, 1 h post-load glucose ≥ 10.0 mmol/L, or 2 h post-load glucose ≥ 8.5 mmol/L. Fetal macrosomia (FM) was defined as birth weight exceeding 4000 g, regardless of gestational age. For in-depth analysis of the amino acid profile in the mother–fetus system and its characteristics according to the presence of FM, all participants were stratified into four subgroups: Subgroup 1 (control)—women without GDM delivering normal-weight infants (n = 18); Subgroup 2—women without GDM delivering macrosomic infants (n = 7); Subgroup 3—women with GDM delivering normal-weight infants (n = 24); Subgroup 4—women with GDM delivering macrosomic infants (n = 13).

### 4.2. Biological Sample Collection

Maternal venous blood samples were collected one day before delivery from the cubital vein into vacuum tubes containing a clot activator for serum separation. Umbilical cord blood samples were collected immediately after delivery and cord clamping by puncture of the umbilical vein into identical tubes. Amniotic fluid samples were obtained during delivery: during elective cesarean section, by intraoperative amniotomy prior to fetal extraction; during vaginal delivery, at the time of amniotomy in the first stage of labor or upon spontaneous rupture of membranes. All samples were centrifuged at 3000 rpm for 15 min to separate cellular components; serum and amniotic fluid were aliquoted into cryovials and stored at −80 °C until analysis.

### 4.3. Amino Acid Concentration Determination

Quantitative determination of free amino acid concentrations in the collected samples was performed using high-performance liquid chromatography-tandem mass spectrometry (HPLC-MS/MS). Sample preparation included protein precipitation with acetonitrile followed by centrifugation, and derivatization of the supernatant using phenylisothiocyanate. Amino acid separation was carried out on a reversed-phase C18 column (150 × 2.1 mm, 3 μm) at 40 °C with gradient elution using a mobile phase consisting of 0.1% formic acid in water and 0.1% formic acid in acetonitrile. Mass spectrometric detection was performed on a triple quadrupole mass spectrometer operating in multiple reaction monitoring mode with electrospray ionization. Calibration was performed using standard amino acid solutions of known concentrations, and isotopically labeled phenylalanine served as the internal standard. Amino acid concentrations were expressed in μmol/L.

### 4.4. Statistical Analysis of Clinical Data

Descriptive statistics for quantitative clinical parameters are presented as medians with interquartile ranges [Q1; Q3], and for categorical clinical parameters as absolute and relative frequencies n (%). Comparison of the four clinical groups was performed using the Kruskal–Wallis test for quantitative variables and the chi-square test for categorical variables. For variables showing statistically significant differences (*p* < 0.05), post hoc analysis was conducted with pairwise group comparisons using Dunn’s test for quantitative variables and Fisher’s exact test for categorical variables, with Bonferroni correction for multiple comparisons. Additionally, comparisons between groups of women with and without GDM was performed by the Mann–Whitney U test for quantitative variables and the chi-square test for categorical variables. Differences were considered statistically significant at *p* < 0.05. To assess test power when comparing two groups, the power of the test was calculated; values below 0.8 were interpreted as insufficient power to detect an effect.

### 4.5. Feature Selection—Clinical Parameters

To identify clinical parameters associated with GDM development, SHAP (Shapley Additive Explanations) values [46] were calculated based on a classification model constructed using the Random Forest algorithm with 1000 decision trees based on age, use of IVF, high risk of preeclampsia, high risk of intrauterine growth restriction, smoking, resus, pre-pregnancy BMI, presence of DM of relatives, presence and type of allergy, GDM in anamnesis, number of pregnancies, number of deliveries, and presence of gynecological disease, metabolic disease and pregnancy events during the first trimester [47]. Parameters with a mean absolute SHAP value of at least half the maximum value were considered to have a potential association with GDM. Similarly, to analyze associations between clinical parameters (age, use of IVF, high risk of preeclampsia, high risk of intrauterine growth restriction, smoking, resus, pre-pregnancy BMI, presence of DM of relatives, presence and type of allergy, GDM in anamnesis, number of pregnancies, number of deliveries, presence of gynecological disease, metabolic disease and pregnancy events during the pregnancy, presence of GDM and GDM-therapy type) and newborn birth weight, SHAP values based on a Random Forest regressor were used, followed by parameter selection according to the same criterion.

### 4.6. Aminoacids Profiling

Variations in amino acid profile were evaluated by principal component analysis using autoscaling. Influence of an amino acid on a principal component was labeled as large in the case of an absolute value of projection more than 0.2. To identify relationships between amino acid levels and quantitative clinical parameters of the women (age, pre-pregnancy and pre-delivery BMI, weight gain during pregnancy, gestation age of delivery, blood biochemical parameters before delivery, urine total protein and newborn weight), correlation analysis was performed using Spearman’s rank correlation coefficient. The threshold for statistical significance was set at *p* < 0.05. Also, levels of amino acid of biofluids from patients with GDM were compared in cases with and without insulin therapy. Tests were performed by Mann–Whitney U test with a statistical significance threshold of *p* < 0.05.

### 4.7. Analysis of Amino Acid Markers

To assess the association between amino acid levels and the presence of GDM, the Mutual Information Index [48] was calculated. Amino acids with an index value of at least half the maximum were considered potential markers. Additionally, the probability of sample distribution overlap was assessed using the Mann–Whitney U test, and the fold change (ratio of median values) and power of test were calculated. The final set of markers included amino acids that were identified as potential based on the Mutual Information Index and had a *p*-value < 0.05 or a fold change greater than 2. To assess the association of amino acid levels with GDM and the development of macrosomia, SHAP values based on Random Forest were also calculated. Amino acids with a mean absolute SHAP value of at least half the maximum calculated value were considered markers.

### 4.8. Metabolic Pathway Enrichment Analysis

Assessment of metabolic pathway enrichment with markers discriminating between comparison groups was performed using the online tool MetaboAnalyst (version 5.0) [49] based on the Small Molecule Pathway Database (SMPDB) [50]. Lists of amino acids identified as markers for each biological compartment and for the combination of GDM with macrosomia were used for analysis. The significance threshold was set based on an unadjusted *p*-value < 0.05.

Statistical data processing was performed using the R programming language (version 4.3.1, Vienna, Austria) [51] with the following packages: DescTools 0.99.60 [52], pwr 1.3-0 [53], effsize 0.8.1 [54], dunn.test 1.3.6 [55], and jgsbook 1.0.7 [56]. To identify features associated with the development of GDM and fetal weight were used with the following packages: praznik 11.0.0 [57], ranger 0.17.0 [58], and kernelshap 0.7.0 [59]. Results were visualized using the pheatmap 1.0.13 [60] and ggplot2 3.5.2 [61].

## 5. Conclusions

The results of this study provide novel insights into the pathogenesis of GDM while offering translational potential for clinical practice. The identified amino acid signatures represent promising candidates for the development of early pregnancy prognostic models to assess the risk of GDM and its complications, including fetal macrosomia. Implementation of such models could facilitate timely dietary and lifestyle modifications and, in the longer term, enable targeted metabolic interventions aimed at restoring amino acid homeostasis and improving perinatal outcomes.

## Figures and Tables

**Table 1:** Concentration of maternal serum amino acids—potential marker of GDM, statistical significance of alteration, fold change, power of test.

Amino Acid	No GDM	GDM	FC	*p*	Power
1-Methylhistidine	7.4 × 10^2^ (6.6 × 10^2^; 8.0 × 10^2^)	6.0 × 10^2^ (5.1 × 10^2^; 7.0 × 10^2^)	0.80	<0.001	0.81
γ-Aminobutyric acid	9.7 × 10^−2^ (9.5 × 10^−3^; 1.5 × 10^−1^)	0 (0; 2.7 × 10^−2^)	<0.001	<0.001	0.88
Glycine	1.4 × 10^2^ (5.5 × 10^1^; 1.7 × 10^2^)	6.2 × 10^1^ (1.1 × 10^1^; 9.5 × 10^1^)	0.44	0.003	0.96
Lysine	2.8 × 10^3^ (2.4 × 10^3^; 3.2 × 10^3^)	2.2 × 10^3^ (1.7 × 10^3^; 2.4 × 10^3^)	0.76	0.001	0.96
Tryptophan	1.3 × 10^2^ (1.1 × 10^2^; 1.5 × 10^2^)	1.1 × 10^2^ (9.7 × 10^1^; 1.3 × 10^2^)	0.88	0.07	0.50

**Table 2:** Concentration of umbilical cord serum amino acids—potential marker of GDM, statistical significance of alteration, fold change, power of test.

Amino Acids	No GDM	GDM	FC	*p*	Power
Alanine	1.1 × 10^3^ (6.0 × 10^2^; 1.8 × 10^3^)	2.4 × 10^1^ (0; 3.5 × 10^2^)	0.02	<0.001	0.98
Asparagine	5.6 × 10^1^ (5.0 × 10^1^; 6.4 × 10^1^)	2.4 × 10^1^ (1.4 × 10^1^; 3.1 × 10^1^)	0.42	<0.001	1.00
Aspartic acid	9.2 × 10^1^ (6.7 × 10^1^; 1.1 × 10^2^)	4.8 × 10^1^ (2.9 × 10^1^; 5.9 × 10^1^)	0.51	<0.001	0.99
β-Alanine	5.9 × 10^−2^ (1.2 × 10^−2^; 0.1.1 × 10^−1^)	0 (0; 0)	<0.001	<0.001	0.93
Citrulline	1.4 × 10^1^ (1.1 × 10^1^; 1.6 × 10^1^)	1.1 × 10^1^ (1.0 × 10^1^; 1.3 × 10^1^)	0.82	0.005	0.80
γ-Aminobutiric acid	4.0 × 10^−1^ (2.8 × 10^−1^; 4.5 × 10^−1^)	2.9 × 10^−2^ (1.1 × 10^−2^; 8.7 × 10^−2^)	0.07	<0.001	1.00
Glutamic acid	2.3 × 10^2^ (2.2 × 10^2^; 2.5 × 10^2^)	1.6 × 10^2^ (1.4 × 10^2^; 1.8 × 10^2^)	0.68	<0.001	1.00
Glutamine	6.6 × 10^4^ (5.6 × 10^4^; 7.4 × 10^4^)	2.9 × 10^4^ (2.6 × 10^4^; 3.5 × 10^4^)	0.44	<0.001	1.00
Glycine	3.07 × 10^2^ (2.2 × 10^2^; 4.0 × 10^2^)	8.0 × 10^1^ (5.0 × 10^1^; 1.1 × 10^2^)	0.26	<0.001	1.00
Methionine	9.9 × 10^1^ (7.4 × 10^1^; 1.3 × 10^2^)	4.5 × 10^1^ (2.7 × 10^1^; 7.1 × 10^1^)	0.46	<0.001	0.97
Proline	8.8 × 10^3^ (8.5 × 10^3^; 1.0 × 10^4^)	7.2 × 10^3^ (6.0 × 10^3^; 8.7 × 10^1^)	0.81	0.001	0.96

**Table 3:** Concentration of amniotic fluid amino acids—potential marker of GDM, statistical significance of alteration, fold change, power of test.

Amino Acid	No GDM	GDM	FC	*p*	Power
1-Methylhistidine	2.5 × 10^2^ (2.1 × 10^2^; 2.8 × 10^2^)	3.1 × 10^2^ (2.5 × 10^2^; 3.9 × 10^2^)	1.21	0.01	0.50
Histidine	4.4 × 10^1^ (2.7 × 10^1^; 7.0 × 10^1^)	4.1 × 10^1^ (3.0 × 10^1^; 6.7 × 10^1^)	0.92	0.91	0.07
Lysine	2.2 × 10^3^ (2.0 × 10^3^; 2.5 × 10^3^)	3.0 × 10^3^ (2.4 × 10^3^; 3.4 × 10^3^)	1.34	0.004	0.89
Threonine	1.1 × 10^2^ (9.2 × 10^1^; 1.7 × 10^2^)	9.9 × 10^1^ (7.9 × 10^1^; 1.4 × 10^2^)	0.87	0.19	0.26

## Data Availability

The original contributions presented in this study are included in the article/Supplementary Materials. Further inquiries can be directed to the corresponding author.

## Supplementary Materials

The following supporting information can be downloaded at https://www.mdpi.com/article/10.3390/ijms27083346/s1.

## Abbreviations

AA	Amino Acid(s)
ACOG	American College of Obstetricians and Gynecologists
AF	Amniotic Fluid
BMI	Body Mass Index
FIGO	International Federation of Gynecology and Obstetrics
FM	Fetal Macrosomia
GABA	Gamma-Aminobutyric Acid
GDM	Gestational Diabetes Mellitus
HAPO	Hyperglycemia and Adverse Pregnancy Outcome
HPLC-MS/MS	High-Performance Liquid Chromatography-Tandem Mass Spectrometry
IVF	In Vitro Fertilization
OR	Odds Ratio
PE	Preeclampsia
SHAP	Shapley Additive Explanations
SMPDB	Small Molecule Pathway Database
