# Supplementary material for: Amino Acid Dysregulation in the Mother–Fetus Unit: Multi-Compartment Metabolomic Signatures of Gestational Diabetes Mellitus and Fetal Macrosomia
Source: Int J Mol Sci. 2026 Apr 8;27(8):3346. doi: 10.3390/ijms27083346 (PMC13116625; doi:10.3390/ijms27083346)
Supplement: Supplementary file 1 [file ijms-27-03346-s001.zip › ijms-4206618-supplementary.pdf]

Supplementary Information

Amino Acid Dysregulation in the Mother–Fetus Unit: Multi-Compartment Metabolomic Signatures of Gestational Diabetes Mellitus and Fetal Macrosomia

Natalia A. Frankevich [1,*], Alisa O. Tokareva [1], Anna A. Derenko [1], Vitaliy V. Chagovets [1], Anastasia V. Novoselova [1], Vladimir E. Frankevich [1,2] and Gennadiy T. Sukhikh [1,3]

[1] National Medical Research Center for Obstetrics, Gynecology and Perinatology Named After Academician V.I. Kulakov of the Ministry of Healthcare of Russian Federation, 117997 Moscow, Russia; a_tokareva@oparina4.ru (A.O.T.); a_derenko@oparina4.ru (A.A.D.); v_chagovets@oparina4.ru (V.V.C.); a_novoselova@oparina4.ru (A.V.N.); v_frankevich@oparina4.ru (V.E.F.); g_sukhikh@oparina4.ru (G.T.S.)

[2] Laboratory of Translational Medicine, Siberian State Medical University, 634050 Tomsk, Russia

[3] Department of Obstetrics, Gynecology, Perinatology and Reproductology, Institute of Professional Education, Federal State Autonomous Educational Institution of Higher Education, I.M. Sechenov First Moscow State Medical University of the Ministry of Health of the Russian Federation, 119991 Moscow, Russia

* Correspondence: n_frankevich@oparina4.ru

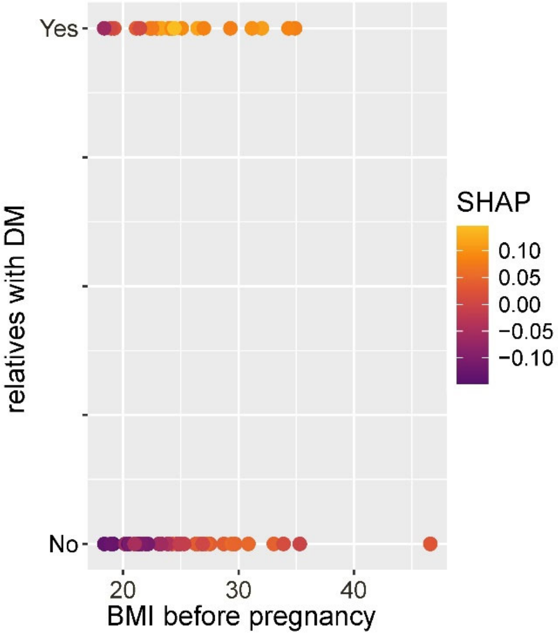

Figure S1. Combined contribution of BMI and family history of diabetes mellitus to the risk of developing GDM. Shapley values of the clinical parameters that most significantly influence the risk of GDM. The color of the points depends on the feature value, ranging from purple (lowest) to yellow (highest).

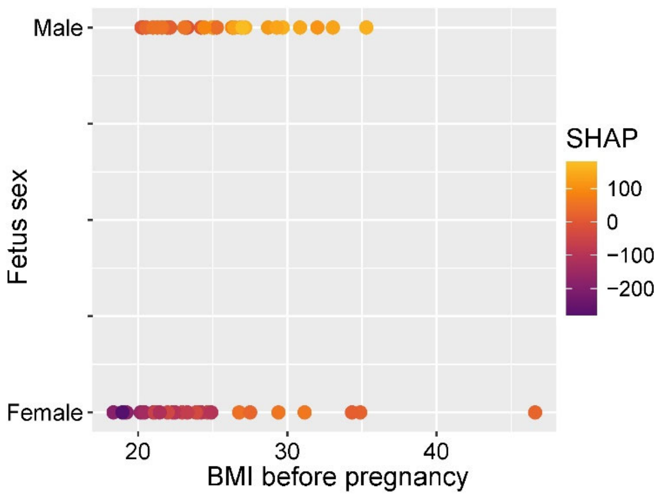

Figure S2. Combined contribution of infant sex and maternal pre-pregnancy BMI to newborn birth weight. The color of the points changes from purple (lowest value) to yellow (highest value).

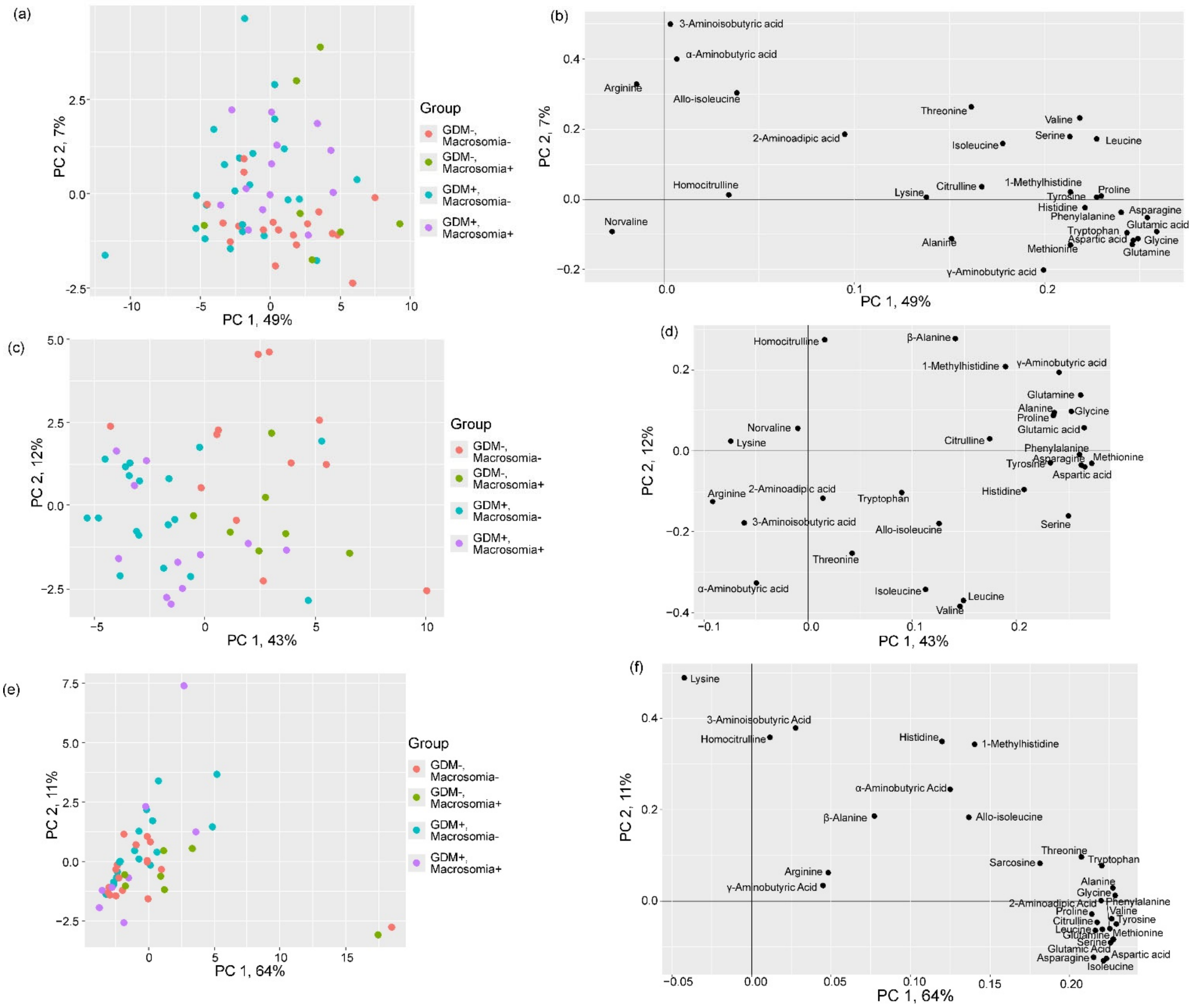

Figure S3. (a) score plot of the maternal venous serum samples in the principal component space; (b) loading plot of the aminoacids from the maternal venous serum samples in the principal component space; (c) score plot of the maternal cord serum samples in the principal component space; (d) loading plot of the aminoacids from the maternal cord serum

samples in the principal component space; (e) score plot of the amniotic fluid samples in the principal component space; (f) loading plot of the aminoacids from the amniotic fluid samples in the principal component space.

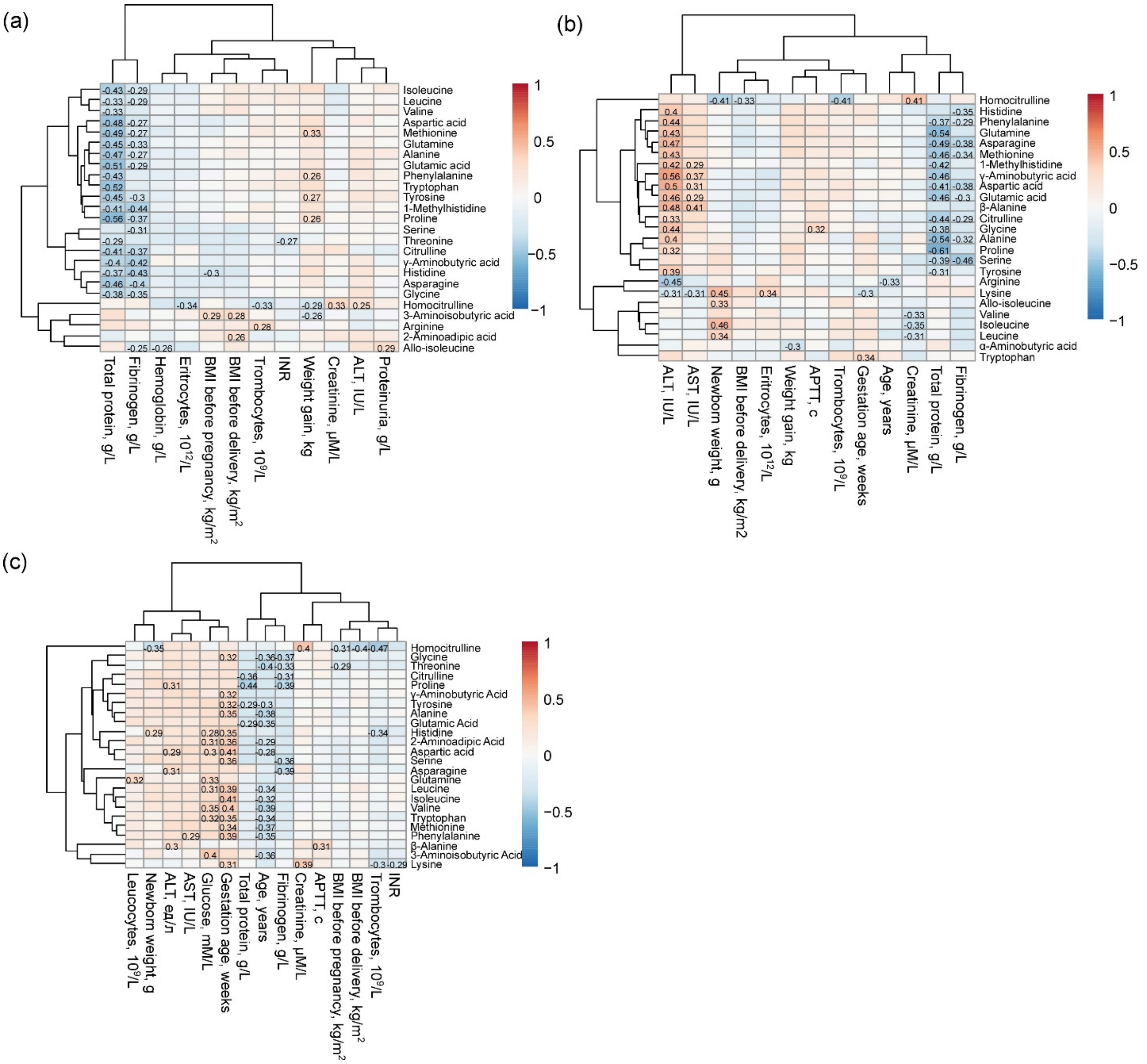

Figure S4. Correlation matrix between maternal and fetus clinical parameters and amino acid levels in case of (a) maternal venous blood serum; (b) maternal cord blood serum; (c) amniotic fluid.

Table S1. Clinical parameters of women without GDM and with developed GDM, probability of difference between values, and test power. Clinical parameters with statistically significant differences are highlighted in bold.

Clinical parameter	GDM -	GDM+	P	Power
Age, years	33 (29; 36)	36 (31; 37)	0.27	0.16
IVF, n (%)	2(8%)	4(11%)	1.00	0.05
High risk of FGR, n (%)	1(4%)	3(8%)	0.91	0.05
High risk of PE, n (%)	**1(4%)**	**10(27%)**	**0.047**	**0.51**
Pre-pregnancy BMI, kg/m²	**22.15 (21.3; 24.65)**	**24.45 (22.32; 28.72)**	**0.03**	**0.59**
BMI category, n (%)	Underweight- 1(4%) Normal weight - 19(76%)	Underweight- 0(0%) Normal weight - 20(54%)	0.09	0.87

	Overweight- 4(16%)<br>Class I obesity - 0(0%)<br>Class II obesity - 1(4%)<br>Class III obesity - 0(0%)	Overweight- 10(27%)<br>Class I obesity - 6(16%)<br>Class II obesity - 0(0%)<br>Class III obesity- 1(3%)		
Gestational weight gain, kg	12 (9; 14)	10 (8; 15)	0.43	0.06
Pathological weight gain, n (%)	0(0%)	5(14%)	0.15	0.30
BMI at delivery, kg/m^2	**27.89 (25.46; 29.38)**	**30.09 (26.7; 32.46)**	**0.03**	**0.60**
Family history of diabetes, n (%)	**4(16%)**	**16(43%)**	**0.048**	**0.51**
Pre-delivery hemoglobin, g/L	127 (118; 133)	127 (120; 134)	0.64	0.09
Pre-delivery erythrocytes, 10^{12}/L	4.24 (4.03; 4.5)	4.38 (4.13; 4.61)	0.26	0.17
Pre-delivery platelets, 10^9/L	243 (219; 267)	227 (199; 276)	0.43	0.12
Pre-delivery leukocytes, 10^9/L	9.19 (8.01; 11.09)	9.62 (8.89; 11.44)	0.33	0.12
Pre-delivery glucose, mmol/L	**4.12 (3.8; 4.7)**	**4.7 (4.1; 5)**	**0.02**	**0.75**
Pre-delivery creatinine, μmol/L	73.5 (67.2; 78.6)	75.6 (70.7; 81.3)	0.15	0.27
Pre-delivery fibrinogen, g/L	5.02 (4.61; 5.43)	5.44 (4.85; 6)	0.08	0.37
Respiratory tract diseases, n (%)	3(12%)	2(5%)	0.65	0.07
Cardiovascular diseases, n (%)	1(4%)	3(8%)	0.91	0.05
Varicose veins, n (%)	2(8%)	5(14%)	0.79	0.06
Thrombosis, n (%)	0(0%)	1(3%)	1.00	0.05
Thrombophilia, n (%)	2(8%)	4(11%)	1.00	0.05
Gastrointestinal tract diseases, n (%)	3(12%)	9(24%)	0.38	0.14
Urinary system diseases, n (%)	None - 23(92%)<br>Cystitis - 1(4%)<br>Pyelonephritis - 1(4%)	None - 32(86%)<br>Cystitis - 5(14%)<br>Pyelonephritis - 0(0%)	0.23	0.40
History of GDM, n (%)	0(0%)	1(3%)	1.00	0.05
PCOS, n (%)	1(4%)	3(8%)	0.91	0.05
Recurrent pregnancy loss, n (%)	0(0%)	1(3%)	1.00	0.05
Number of pregnancies	1 (1; 3)	2 (1; 2)	0.47	0.05
Number of deliveries	1 (1; 2)	1 (1; 2)	0.70	0.05
History of macrosomia, n (%)	0(0%)	6(16%)	0.09	0.39
Dietary therapy, n (%)	**2(8%)**	**33(89%)**	**<0.001**	**1.00**
Insulin therapy, n (%)	**0(0%)**	**12(32%)**	**0.004**	**0.81**
Insulin in 2nd trimester, n (%)	0(0%)	7(19%)	0.06	0.48
Insulin in 3rd trimester, n (%)	**0(0%)**	**12(32%)**	**0.004**	**0.81**
Amniotic fluid level, n (%)	normal - 22(88%)<br>oligohydramnios - 1(4%)<br>polyhydramnios - 2(8%)	normal - 31(84%)<br>oligohydramnios - 4(11%)<br>polyhydramnios - 2(5%)	0.59	0.18
Gestational age at delivery, weeks	39 (38; 40)	39 (38.6; 39.4)	0.83	0.05
Induced labor, n (%)	10(40%)	16(43%)	1.00	0.05
PROM, n (%)	1(4%)	1(3%)	1.00	0.05
Early PROM, n (%)	0(0%)	1(3%)	1.00	0.05

Fetal hypoxia, n (%)	1(4%)	1(3%)	1.00	0.05
Cesarean section, n (%)	None- 12(48%) Planned - 11(44%) Emergency- 2(8%)	None- 16(43%) Planned - 17(46%) Emergency - 4(11%)	0.90	0.07
Maternal hospital stay, days	3 (3; 5)	4 (3; 5)	0.27	0.36
Male fetal sex, n (%)	13(52%)	19(51%)	1.00	0.05
Fetal weight, g	3518 (2824; 4014)	3600 (3238; 4096)	0.37	0.18
Apgar score at 1 minute	8 (8; 8)	8 (8; 8)	0.43	0.13
Apgar score at 5 minutes	9 (9; 9)	9 (9; 9)	1.00	0.05
NICU admission, n (%)	0(0%)	2(5%)	0.65	0.07
Neonatal pathology department admission, n (%)	0(0%)	3(8%)	0.39	0.14
Newborn hospital stay, days	3 (3; 5)	4 (3; 5)	0.49	0.07

Table S2. Clinical parameters across four clinical groups and the probability of concordance between groups. Clinical parameters with statistically significant differences are highlighted in bold.

Clinical parameters	GDM-, normosomia (Group 1)	GDM-, macrosomia (Group 2)	GDM+, normosomia (Group 3)	GDM+, macrosomia (Group 4)	P
Age, years	33 (28.25; 36.75)	32 (31; 34)	36 (28.75; 37)	35 (31; 37)	0.67
IVF, n (%)	1(6%)	1(14%)	3(12%)	1(8%)	0.85
**Pre-pregnancy BMI, kg/m²**	**21.8 (20.8; 23.7)**	**26.2 (22.6; 28.1)**	**24.1 (21.8; 27.0)**	**26.5 (24.5; 29.7)**	**0.02 p_{14} = 0.007**
BMI category, n (%)	Underweight - 1(6%) Normal weight - 15(83%) Overweight- 2(11%) Class I obesity- 0(0%) Class II obesity - 0(0%) Class III obesity - 0(0%)	Underweight- 0(0%) Normal weight - 3(43%) Overweight- 3(43%) Class I obesity- 0(0%) Class II obesity - 1(14%) Class III obesity - 0(0%)	Underweight- 0(0%) Normal weight - 15(62%) Overweight- 5(21%) Class I obesity - 3(12%) Class II obesity- 0(0%) Class III obesity- 1(4%)	Underweight - 0(0%) Normal weight - 5(38%) Overweight- 5(38%) Class I obesity- 3(23%) Class II obesity - 0(0%) Class III obesity- 0(0%)	0.08
Gestational weight gain, kg	12 (9.25; 13)	12 (10; 19)	9.5 (7; 12.25)	12 (10; 23)	0.16
**Pathological weight gain, n (%)**	**0(0%)**	**0(0%)**	**1(4%)**	**4(31%)**	**0.008**
**BMI at delivery, kg/m²**	**26.0 (24.3; 28.5)**	**30.8 (27.5; 31.9)**	**28.4 (25.2; 31.5)**	**30.9 (30.1; 33.4)**	**0.002 p_{14} <0.001**
Family history of diabetes, n (%)	2(11%)	2(29%)	12(50%)	4(31%)	0.07

Pre-delivery hemoglobin, g/L	127 (120.25; 131.5)	128 (115; 135)	127 (120; 132.5)	125 (121; 134)	0.95
Pre-delivery erythrocytes, 10^{12}/L	4.2 (3.98; 4.43)	4.29 (4.14; 4.65)	4.28 (4.09; 4.54)	4.43 (4.38; 4.66)	0.24
Pre-delivery platelets, 10^{9}/L	236 (212; 263)	243 (227; 294)	235 (189; 303)	211 (206; 250)	0.39
Pre-delivery leukocytes, 10^{9}/L	8.8 (8.0; 11.2)	9.6 (8.7; 10.7)	9.9 (9.0; 11.5)	9.2 (8.8; 9.8)	0.63
**Pre-delivery glucose, mmol/L**	**4 (3.56; 4.2)**	**4.72 (4.45; 5.1)**	**4.4 (4.0; 4.8)**	**4.8 (4.6; 5.9)**	**0.003**<br>**p_{14} = 0.005**
Pre-delivery creatinine, μmol/L	76.5 (70.7; 78.0)	67.6 (65.9; 75.1)	78.7 (70.7; 81.7)	75.1 (70.7; 78.8)	0.24
Pre-delivery total protein, g/L	63.9 (61.9; 66.3)	65.7 (64.0; 67.1)	64.8 (63.05; 66.8)	65.6 (63.3; 68.3)	0.87
**Pre-delivery APTT, sec**	**27.3 (25.9; 28.4)**	**28.6 (28.1; 28.8)**	**26.6 (25.1; 27.6)**	**26.1 (25.7; 27.1)**	**0.04**<br>**p_{23}=0.04**<br>**p_{24}=0.03**
Pre-delivery fibrinogen, g/L	5.2 (4.8; 5.5)	4.7 (4.6; 4.9)	5.5 (4.8; 6.2)	5.2 (5.1; 5.5)	0.17
Pre-delivery INR	0.92 (0.88; 0.96)	0.96 (0.94; 0.97)	0.91 (0.89; 0.95)	0.93 (0.91; 0.95)	0.17
Urine protein, g/L	0 (0; 0)	0 (0; 0.15)	0.06 (0; 0.12)	0 (0; 0.1)	0.34
Respiratory tract diseases, n (%)	3(17%)	0(0%)	0(0%)	2(15%)	0.14
Cardiovascular diseases, n (%)	0(0%)	1(14%)	2(8%)	1(8%)	0.55
Anemia, n (%)	2(11%)	0(0%)	1(4%)	0(0%)	0.46
Thrombophilia, n (%)	1(6%)	1(14%)	3(12%)	1(8%)	0.85
Gastrointestinal tract diseases, n (%)	3(17%)	0(0%)	6(25%)	3(23%)	0.50
Urinary system diseases, n (%)	None - 16(89%)<br>Cystitis - 1(6%)<br>Pyelonephritis - 1(6%)	None - 7(100%)<br>Cystitis - 0(0%)<br>Pyelonephritis - 0(0%)	None - 22(92%)<br>Cystitis - 2(8%)<br>Pyelonephritis - 0(0%)	None - 10(77%)<br>Cystitis - 3(23%)<br>Pyelonephritis- 0(0%)	0.40
Hypothyroidism, n (%)	4(22%)	1(14%)	2(8%)	3(23%)	0.56
History of GDM, n (%)	0(0%)	0(0%)	0(0%)	1(8%)	0.28
PCOS, n (%)	1(6%)	0(0%)	2(8%)	1(8%)	0.88
Number of pregnancies	1 (1; 2.75)	1 (1; 2.5)	2 (1; 2)	2 (1; 3)	0.70
Number of deliveries	1 (1; 1.75)	1 (1; 2)	1 (1; 2)	1 (1; 2)	0.86
**History of macrosomia, n (%)**	**0(0%)**	**0(0%)**	**2(8%)**	**4(31%)**	**0.03**

**Dietary therapy, n (%)**	**2(11%)**	**0(0%)**	**23(96%)**	**10(77%)**	**<0.001 p_{13}<0.001 p_{14}=0.005 p_{24}<0.001 p_{34} = 0.03**
**Insulin therapy, n (%)**	**0(0%)**	**0(0%)**	**8(33%)**	**4(31%)**	**0.02**
Amniotic fluid volume, n (%)	Normal - 15(83%) Oligohydramnios - 1(6%) Polyhydramnios - 2(11%)	Normal - 7(100%) Oligohydramnios - 0(0%) Polyhydramnios - 0(0%)	Normal - 21(88%) Oligohydramnios - 3(12%) Polyhydramnios - 0(0%)	Normal - 10(77%) Oligohydramnios - 1(8%) Polyhydramnios - 2(15%)	0.44
Gestational age at delivery, weeks	38.3 (38; 39.9)	40 (39; 40)	39.15 (38.5; 39.42)	39 (39; 39)	0.56
Induced labor, n (%)	6(33%)	4(57%)	10(42%)	6(46%)	0.73
PROM, n (%)	1(6%)	0(0%)	0(0%)	1(8%)	0.54
Early PROM, n (%)	0(0%)	0(0%)	1(4%)	0(0%)	0.66
Fetal hypoxia, n (%)	1(6%)	0(0%)	1(4%)	0(0%)	0.79
Hemorrhage, n (%)	1(6%)	0(0%)	0(0%)	0(0%)	0.48
Cesarean section, n (%)	None - 7(39%) Planned - 9(50%) Emergency - 2(11%)	None - 5(71%) Planned - 2(29%) Emergency - 0(0%)	None - 9(38%) Planned - 12(50%) Emergency - 3(12%)	None - 7(54%) Planned- 5(38%) Emergency - 1(8%)	0.74
Maternal hospital stay, days	4 (3; 5)	3 (3; 3.5)	4 (3; 5)	4 (3; 5)	0.51
**Male fetal sex, n (%)**	**7(39%)**	**6(86%)**	**9(38%)**	**10(77%)**	**0.02**
**Fetal weight, g**	**3107(2638; 3640)**	**4078 (4043; 4243)**	**3369 (2985; 3578)**	**4114 (4096; 4232)**	**<0.001 p_{12}<0.001 p_{23}<0.001 p_{14}<0.001 p_{34}<0.001**
Apgar score at 1 minute	8 (8; 8)	8 (8; 8)	8 (8; 8)	8 (8; 8)	0.29
Apgar score at 5 minutes	9 (9; 9)	9 (9; 9)	9 (9; 9)	9 (9; 9)	0.92
**Resuscitation measures, n (%)**	**0(0%)**	**1(14%)**	**0(0%)**	**0(0%)**	**0.046**
NICU admission, n (%)	0(0%)	0(0%)	1(4%)	1(8%)	0.63

Neonatal pathology department admission, n (%)	0(0%)	0(0%)	2(8%)	1(8%)	0.54
Newborn hospital stay, days	3 (3; 5)	3 (2.5; 4)	4 (3; 5)	4 (3; 5)	0.55

Table S3. Statistical significant of variance of amino acid levels in case of GDM with and without insulintherapy.

Amino acid	Serum	Cord	Amniotic fluid
1-Methylhistidine	0.59	0.95	0.61
2-Aminoadipic acid	0.19	1.00	0.14
3-Aminoisobutyric acid	0.26	0.21	0.64
Alanine	0.76	0.13	0.50
Allo-isoleucine	1.00	0.98	0.33
α-Aminobutyric acid	0.86	0.76	0.20
Arginine	0.11	0.57	0.84
Asparagine	0.90	0.57	0.56
Aspartic acid	0.99	0.84	0.11
β-Alanine	NA	0.64	0.20
Citrulline	0.29	0.51	0.46
γ-Aminobutyric acid	0.92	0.98	0.53
Glutamic acid	0.96	0.64	0.33
Glutamine	0.45	0.57	0.24
Glycine	0.58	0.45	0.33
Histidine	0.66	0.54	1.00
Homocitrulline	0.88	0.19	0.57
Isoleucine	0.84	0.23	0.30
Leucine	0.74	0.67	1.00
Lysine	0.52	0.60	0.84
Methionine	0.75	0.84	0.66
Norvaline	0.58	0.64	NA
Phenylalanine	0.99	0.77	0.66
Proline	0.74	0.14	0.46
Sarcosine	NA	NA	0.57
Serine	0.24	0.70	0.14
Threonine	0.30	0.91	0.33
Tryptophan	0.99	0.38	0.57
Tyrosine	0.88	0.10	0.33
Valine	0.85	0.60	0.50

Table S4. Changes in amino acid levels across three biological matrices (maternal serum, cord serum, and amniotic fluid) in GDM.

**Amino Acid / Metabolite**	Compartment	Change in GDM (overall)	Features in GDM + Macrosomia

**Glycine**	Maternal serum	Decrease (GDM marker)	No specific differences identified
	Cord serum	Decrease (GDM marker)	Lower levels characterize GDM cases with normosomia (i.e., in macrosomia, the level may be relatively higher than in GDM without macrosomia, but still below control levels)
**1-Methylhistidine**	Maternal serum	Decrease (GDM marker)	Control group marker (normal); decreased in GDM
	Amniotic fluid	Increase (GDM marker)	Elevated in the GDM group delivering a normal-weight infant (in contrast to maternal blood)
**γ-Aminobutyric acid (GABA)**	Maternal serum	Decrease (GDM marker)	No specific differences identified
	Cord serum	Decrease (GDM marker)	Lower levels characterize GDM cases with normosomia
**Lysine**	Maternal serum	Decrease (GDM marker)	Control group marker; specific decrease in GDM with delivery of a normal-weight infant
	Cord serum	(Not a primary GDM marker, but significant for outcome)	Higher level characterizes GDM cases with macrosomia (differentiates normo- and macrosomia)
	Amniotic fluid	Increase (GDM marker)	Elevated in the GDM group delivering a normal-weight infant; in GDM with macrosomia — low levels
**Tryptophan**	Maternal serum	Decrease (GDM marker)	No specific differences identified
**Glutamine**	Cord serum	Decrease (GDM marker)	Control group marker (high in normal, low in GDM)
**Asparagine**	Cord serum	Decrease (GDM marker)	Control group marker; in GDM with macrosomia — low levels (in amniotic fluid)
**Methionine**	Cord serum	Decrease (GDM marker)	No specific differences identified
**Aspartic acid**	Cord serum	Decrease (GDM marker)	No specific differences identified
**Glutamic acid**	Cord serum	Decrease (GDM marker)	No specific differences identified
β-Alanine	Cord serum	Decrease (GDM marker)	No specific differences identified
Proline	Cord serum	Decrease (GDM marker)	Higher level characterizes GDM cases with macrosomia (differentiates normo- and macrosomia)
Citrulline	Cord serum	Decrease (GDM marker)	Lower levels characterize GDM cases with normosomia
Alanine	Cord serum	Decrease (GDM marker)	No specific differences identified
Allo-isoleucine	Cord serum	(Not a primary GDM marker, but	Higher levels characterize GDM cases with macrosomia

		significant for outcome)	
Leucine (and isoleucine)	Cord serum	(Not a primary GDM marker, but significant for outcome)	Higher levels characterize GDM cases with macrosomia (isoleucine has a direct correlation with fetal weight)
Threonine	Amniotic fluid	GDM marker (direction not specified)	No specific differences identified
Histidine	Amniotic fluid	GDM marker (direction not specified)	High levels are characteristic of GDM with delivery of a normal-weight infant
Homocitrulline	Amniotic fluid	(Not a primary GDM marker, but significant for outcome)	Low levels are characteristic of GDM with delivery of a normal-weight infant; has an inverse correlation with fetal weight and maternal BMI

Table S5. Biological interpretation of changes in key amino acid levels across different biological matrices in GDM.

Amino Acid	Integrated Biological Interpretation
Glycine	Systemic glycine deficiency. Glycine possesses anti-inflammatory and immunomodulatory properties and participates in the synthesis of glutathione (an antioxidant) and purines. Its decrease in both the systemic (maternal) and fetoplacental (cord) compartments indicates a general shift in metabolism toward chronic low-grade inflammation and oxidative stress, characteristic of insulin resistance and GDM.
1-Methylhistidine	Marker of muscle tissue catabolism. 1-Methylhistidine is a breakdown product of the muscle protein actin. Its decrease in maternal blood may indirectly indicate impaired protein metabolism in the mother. An increase in amniotic fluid is an alarming sign, potentially indicating increased protein catabolism in the fetus in response to metabolic stress (hyperglycemia) or changes in amniotic fluid composition due to fetal polyuria.
γ-Aminobutyric acid (GABA)	Disruption of GABAergic regulation. GABA is the main inhibitory neurotransmitter but also performs metabolic functions in peripheral tissues, including modulation of insulin secretion by pancreatic β-cells. Its decrease may reflect islet cell dysfunction and impaired metabolic signaling in both the mother and the fetoplacental unit.
Lysine	Imbalance in essential amino acid metabolism. Lysine is an essential amino acid. Its decrease in maternal blood may indicate altered consumption or distribution. The increase in AF requires cautious interpretation: it may result from altered fetal renal excretion or impaired transport across fetal membranes.
Glutamine	Key indicator of placental transport and fetal energy supply. Glutamine is a critical energy source for enterocytes, immune cells, and proliferating fetal tissues, and is a precursor to glutamate and GABA. Its decrease in cord blood provides direct evidence of impaired

Amino Acid	Integrated Biological Interpretation
	placental transport or increased utilization of this amino acid by the fetus under stress conditions, potentially limiting growth and development.
Asparagine	Associated disruption of amide metabolism. Often coupled with glutamine. Involved in the synthesis of purines and pyrimidines (nucleotides), essential for rapid fetal cell division. Its decrease indicates broader impairments in the supply of substrates for fetal growth.
Citrulline	Disruption of the urea cycle and NO synthesis. Citrulline is an intermediate metabolite of the urea cycle and a precursor to arginine, from which nitric oxide (NO)—a potent vasodilator—is synthesized. Its decrease may reflect endothelial dysfunction and impaired vascular tone in the fetoplacental bed, characteristic of GDM complications.
Tryptophan	Modulation of immune response and the serotonergic system. Tryptophan is a precursor to serotonin (the "feel-good hormone") and kynurenine (an immunoregulator). Its imbalance is associated with the risk of depression and immune dysfunction. Disruption of its metabolism in GDM may contribute to the pathogenesis of associated complications.
